# Supplementary material for: Shaping the topology of folding pathways in mechanical systems
Source: Nat Commun. 2018 Oct 16;9:4303. doi: 10.1038/s41467-018-06720-1 (PMC6191449; doi:10.1038/s41467-018-06720-1)
Supplement: Supplementary file 1 — Supplementary Information [file 41467_2018_6720_MOESM1_ESM.pdf]

Supplementary Information

Shaping the topology of folding pathways in mechanical systems

Stern et al.

Supplementary Figure 1

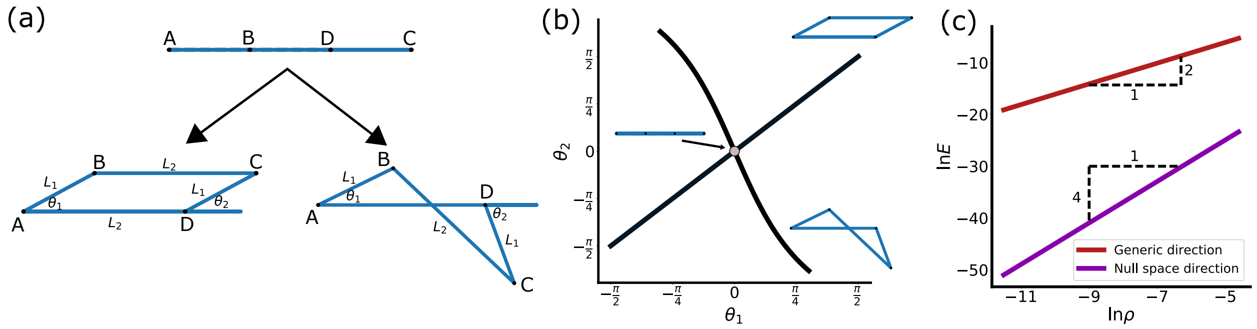

Supplementary Figure 1. **Modes of the 4-bar linkage**. (a) A stiff parallelogram type 4-bar linkage bifurcates from the top 'flat' state into two distinguishable motions, (b) The two motions are defined by the relations imposed on angles θ_1, θ_2, (c) The two special zero-energy motions span a linearized null space, configurations in which scale quartically with the distance form the flat state $E \sim \rho^4$. In contrast, random configurations of the linkage have an energy that scales quadratically with this distance $E \sim \rho^2$.

Supplementary Figure 2

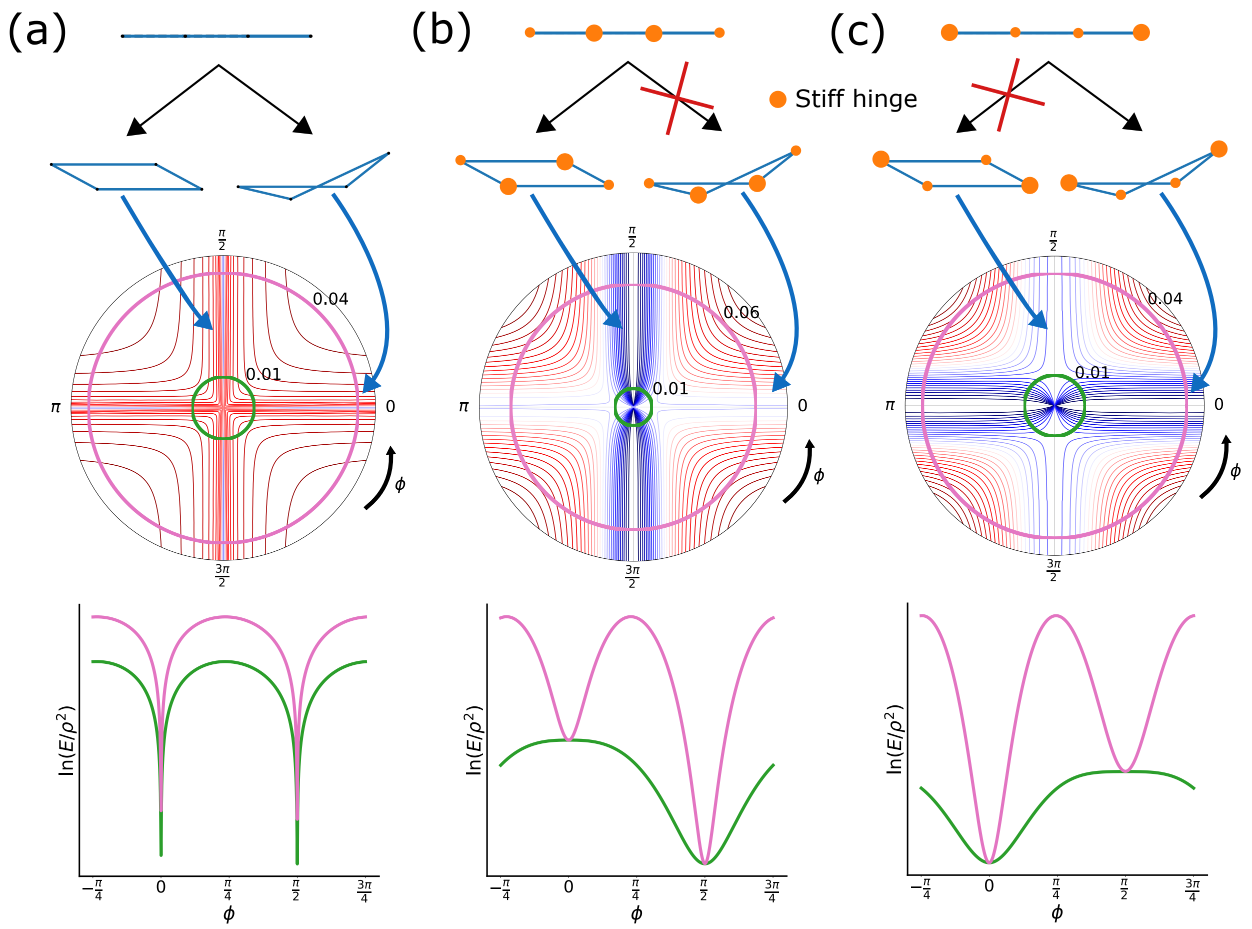

Supplementary Figure 2. **Each of the linkage modes can be lifted with proper hinge stiffness values**. (a) With freely rotating hinges, the two zero-modes bifurcate at the flat state in the center of the landscape diagram, whose radius corresponds to ρ, and angle ϕ is the mixing angle of the two zero-modes spanning the linearized null space (contours represent scaled energy E/ρ^2), (b) Choosing the two hinges denoted by large orange circles to be stiffer than the other two, the right mode is lifted, making only the left mode continuously accessible from the flat state, shown as a continuous blue valley starting at $\rho = 0$. The right mode is regained at a larger radius $\rho > \rho_c$, as seen in the contour curvature, (c) By making the other two hinges stiffer, the left mode is lifted near the flat state.

Supplementary Figure 3

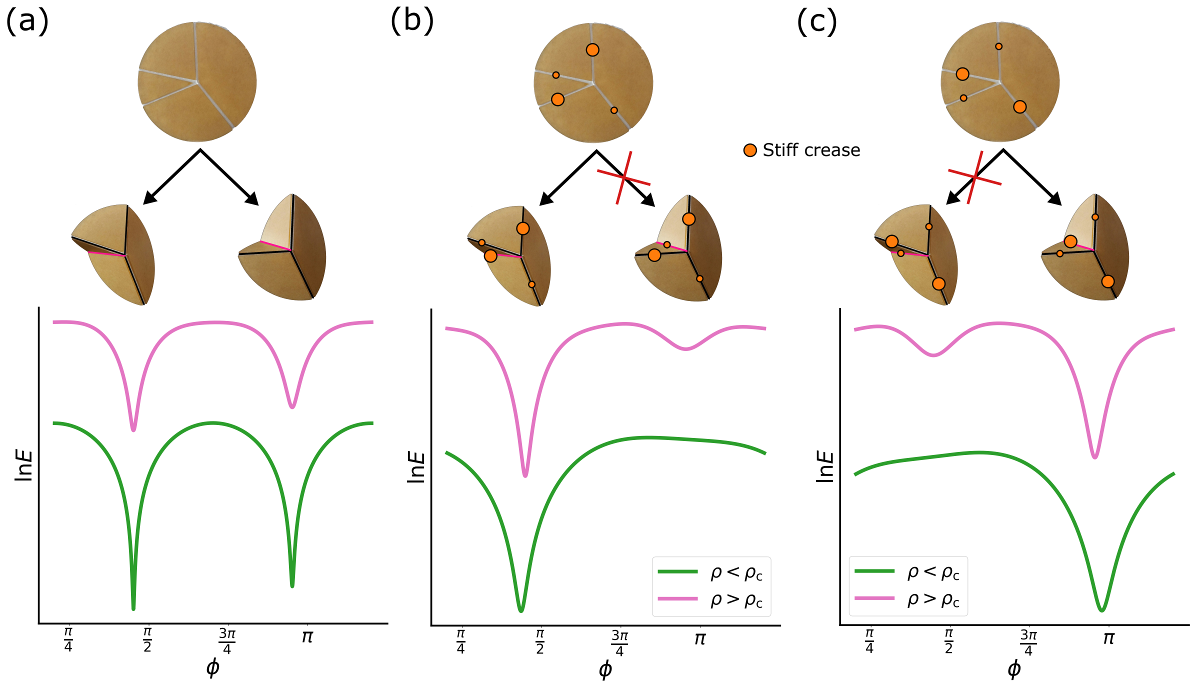

Supplementary Figure 3. **Lifting modes in origami 4-vertices**. Just like in the 4-bar linkage example, each of the two zero-modes of the origami 4-vertex can be lifted given a proper crease stiffness profile. (a) The modes of a vertex with perfectly soft creases bifurcate at the flat state, so that two minima appear in the linearized null space for any value of the folding magnitude ρ (ϕ is the mixing angle of two vectors spanning the linearized null space), (b) The heterogeneous crease stiffness described here, with stiffer creases denoted by larger orange circles, lifts the right mode, so that only the left mode is continuously accessible from the flat state (the right mode is again regained at larger folding values $\rho > \rho_c$), (c) Changing the crease stiffness profile as shown, we can instead lift the left mode near the flat state.

Supplementary Figure 4

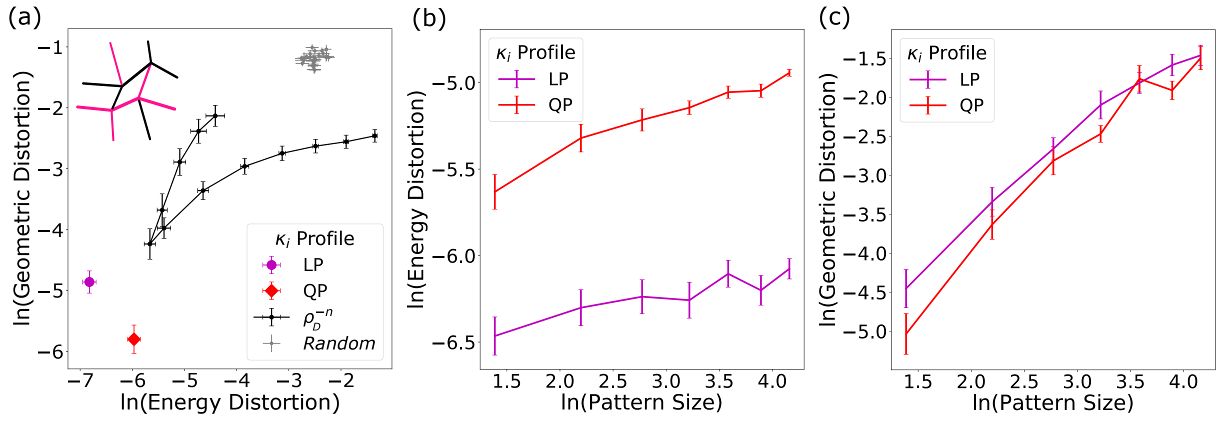

Supplementary Figure 4. **Performance of Linear and Quadratic Programming stiffness selection protocols for large origami patterns**. a) LP and QP stiffness optimize energetic and geometric distortion of the designed folding motion for quad patterns. LP and QP stiffness profiles improve these distortions by orders of magnitude compared to random profiles. b) Larger patterns with many vertices are harder to optimize, with designed motion energy scaling as soft power laws with system size. c) Geometric distortion grows with system size.

Supplementary Figure 5

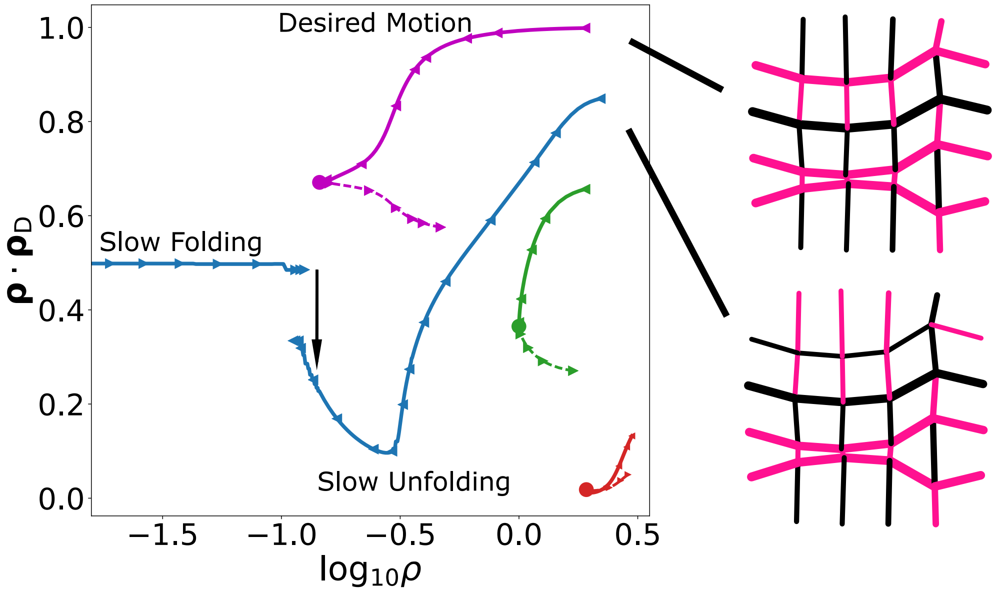

Supplementary Figure 5. **A pattern for which slow folding with LP stiffness profile fails**. The low-ρ unique minimum, rather then continuously connecting to the desired state, terminates in a saddle-node bifurcation and discontinuously jumps to a distractor folded state.

Supplementary Note 1 - 4-bar linkage

The 4-bar linkage [1, 2] discussed in the main text is composed of 2 pairs of rods with lengths L_1, L_2 (Supplementary Figure 1(a)). For simplicity, let us assume that points A and D are held fixed in space with distance L_2 between them. Points B and C are allowed to move in a plane, with an energy cost associated with changing the lengths of the three rods from their relaxed state $E = \frac{1}{2}K\Delta L^2$ (alternatively, energy cost can arise from bending the three rods from their relaxed straight configuration).

This system is a one degree-of-freedom mechanism, as there are 3 constraints (rod lengths) imposed on 4 variables (x, y positions of nodes B, C). As such, configurations satisfying the constraints define one-dimensional motions. These particular cases are fully described in terms of the angles θ_1, θ_2 between rods $L_{\mathrm{AB}}, L_{\mathrm{CD}}$ and the x-axis. When accounting for the non-linearity of the constraints, one finds two one-dimensional solutions (Supplementary Figure 1(b)) for the system motions given by [3]

$$\begin{aligned} \theta_2^\alpha &= \theta_1 \\ \theta_2^\beta &= \cos^{-1}\Big\{\frac{[1+(\frac{L_2}{L_1})^2]\cos\theta_1 - 2\frac{L_2}{L_1}}{[1+(\frac{L_2}{L_1})^2] - 2\frac{L_2}{L_1}\cos\theta_1}\Big\} \end{aligned} \tag{1}$$

The two solutions share a configuration $\theta_1 = 0 \rightarrow \theta_2 = 0$. Consider this special 'flat state' $\theta_1 = \theta_2 = 0$, for which the node positions are $\mathbf{P}_0 = [x_{\mathrm{B}} = L_1, y_{\mathrm{B}} = 0, x_{\mathrm{C}} = L_1 + L_2, y_{\mathrm{C}} = 0]$. Let us denote the distance of a configuration from the flat state by $\rho \equiv ||\boldsymbol{P} - \boldsymbol{P}_0||$. When constraints are allowed to be broken by compression (or bending) of the rods, allowing nonzero energy configurations in the full 4-dimensional $\boldsymbol{\rho}$-space, the configuration energy generically scales as $E \sim K(\boldsymbol{P} - \boldsymbol{P}_0)^2 \sim \rho^2$. However, close to the flat state the two special zero-energy motions span a linearized null space in which the energy scales more softly $E_{\mathrm{NS}} \sim K(\boldsymbol{P} - \boldsymbol{P}_0)^4 \sim \rho^4$ (Supplementary Figure 1(c)). To see this, note that to lowest order in ρ, the two zero-energy motions are composed of just vertical motions of points B, C:

$$\boldsymbol{v}_\pm = \frac{1}{\sqrt{2}}[0, 1, 0, \pm 1]$$

These vertical motions are small compared to rod lengths, and the Pythagorean theorem tells us that the energy difference due to rod extension vanishes to quadratic order. Main

text Figure 1 shows the energy of configurations in the null space for given magnitudes of the vector $\boldsymbol{P} - \boldsymbol{P}_0$.

As the null space energy of the system scales like ρ^4, we show that placing heterogeneous quadratic torsional springs on the hinges raises the energy of one motion more than the other. This facilitates the removal of the flat state bifurcation, and the mechanical preference of just one of the two motions. Let us first define the energy model for the 4-bar linkage:

$$E(\boldsymbol{\rho}) \equiv \frac{1}{2} K \sum_{i \in \text{bars}} \Delta L_i^2 + \frac{1}{2} \sum_{j \in \text{hinges}} \kappa_j \theta_j^2, \tag{2}$$

With torsional springs κ_j attached to each of the four hinges, such that the springs are relaxed at the flat state. With stiff hinges, the energy of all configurations now scale as ρ^2 (including those in the linearized null space). However, as different hinges have different stiffness values, it is possible to raise the energy of different configurations to differing extents. If all hinges are free (Supplementary Figure 2(a)), we again have the two zero-modes established before. The middle row shows the scaled null space energy E/ρ^2, with radius ρ and angle ϕ is the mixing angle of the two zero-modes spanning the 2-dimensional null space:

$$\boldsymbol{\rho} = \rho(\boldsymbol{v}_+ \sin\phi + \boldsymbol{v}_- \cos\phi)$$

The energy is scaled by ρ^2 so that the low energy paths in the landscape are clearly visible in the contour topography as blue valleys between red ridges.

Let us now choose a heterogeneous hinge stiffness profile as seen in Supplementary Figure 2(b). Here, large circles correspond to stiff hinges with stiffness values $\kappa_1 = 10^{-4}K$, while small circles denote softer hinges with $\kappa_2 = 10^{-6}K$. With this choice we find that one of the original zero-modes is lifted up to a distance from the flat state $\rho_c \sim \sqrt{\kappa_1/K} = 10^{-2}$. At $\rho \sim \rho_c$, the lifted mode returns by a saddle-node bifurcation, and is clearly seen as a stable minimum in larger radii. By switching the locations of the stiff and soft hinges, we find that the other zero-mode is lifted near the flat state (Supplementary Figure 2(c)). As in the other choice of hinge stiffness value, the lifted zero mode returns via a saddle-node bifurcation at $\rho \sim \rho_c$.

In general, when heterogeneous stiffness profiles are applied to the hinges, a critical crossover value arises $\rho_c \sim \sqrt{\tilde{\kappa}/K}$ ($\tilde{\kappa}$ the dominant hinge stiffness scale), where the stiff hinge energy is comparable to the intrinsic energy. We find that one minimum exists in the

energy landscape at distances $\rho \ll \rho_c$. The unique minimum corresponds to one of the two zero-modes of the original system, and continuously connects to it when ρ is increased. The other zero-mode of the system is always regained at $\rho \sim \rho_c$ via a saddle-node bifurcation. If a random heterogeneous stiffness profile is chosen, the zero-mode that survives close to the flat state is decided by which pair of hinges is stiffer, as described by Supplementary Figure 2(b-c).

Finally, given a fluctuation energy scale ϵ, effectively removing the bifurcation requires the energy barriers when the second minimum is regained to be larger than ϵ. This requirement establishes a lower bound on the hinge stiffness $\tilde{\kappa} > \sqrt{\epsilon K}$.

Supplementary Note 2 - Origami energy model

4-Vertex energy

Any origami vertex embedded in 3-dimensional space gives rise to 3 constraints relating the dihedral angles by which the different creases fold (i.e. angles between faces) [4, 5]. These constraints are derived for any vertex by requiring that the vertex faces do not bend when folded. Consider a small disk surrounding the vertex, whose configurations is defined by the folding angles ρ_i and the in-plane angles between the creases α_i. If the folded vertex faces do not bend anywhere, it is possible to trace the edge of the disk around the vertex and return to the starting point using simple 3d rotations. The tracing motion around the vertex consists of alternating rotations with the circular section angle about the central axis of the vertex α_i, and then rotating about the dihedral angle ρ_i. This is done for each crease, until one returns to the original position on the vertex. If one orients the current vertex face such that the face occupies the xy-plane and the crease is on the x-axis, these two rotation matrices are given by

$$R_i = \begin{pmatrix} 1 & 0 & 0 \\ 0 & \cos\rho_i & -\sin\rho_i \\ 0 & \sin\rho_i & \cos\rho_i \end{pmatrix} \begin{pmatrix} \cos\alpha_i & -\sin\alpha_i & 0 \\ \sin\alpha_i & \cos\alpha_i & 0 \\ 0 & 0 & 1 \end{pmatrix}. \tag{3}$$

In general $R_i = A_i B_i$ where A_i is a rotation matrix about an axis along crease i by angle ρ_i, while B_i is a rotation matrix about an axis perpendicular to face i by angle α_i.

The condition that the vertex faces do not bend becomes

$$\prod_i R_i = I, \tag{4}$$

where the product is taken over all creases and faces i, and I is the 3×3 identity matrix. Supplementary Equation (4) can be shown to be equivalent to 3 independent equations for the off-diagonal upper triangle of the matrix. Crucially, these are 3 non-linear constraints relating the folding values ρ_i around the vertex. The 4-vertex is a one degree-of-freedom object, as 3 equation relate its 4 folding angles.

Similarly to the linkage discussed above, the fact that an origami 4-vertex can only support one-dimensional motions does not imply that only one such motion exists. A single 4-vertex is known to have two zero-energy folding motions that meet at the flat, unfolded state. These special motions exactly satisfy the three vertex constraints.

Near the flat state, all $\rho_i \approx 0$, and the matrices of equations (3-4) are approximately $2d$ rotations about an axis perpendicular to the flat vertex. One vertex constraint thus becomes degenerate, and a linearized 2d null space emerges. The preceding considerations apply to all vertices constructing the pattern.

The constraint equations can be expanded about the flat state $\boldsymbol{\rho} = 0$:

$$T_a(\boldsymbol{\rho}) = C_a^i \rho_i + D_a^{ij} \rho_i \rho_j + \dots \tag{5}$$

We take the energy of the vertex to be,

$$E_{\text{Vertex}} = \frac{1}{2} \kappa_{\text{f}} \sum_a T_a^2$$

with κ_{f} a face bending modulus [6–9]. Thus our energy is a measure of the violation of vertex constraints in Supplementary Equation 4. For thin sheets, violation of vertex constraints manifests as bending of vertex faces since stretching is energetically expensive compared to bending [10].

With this definition of vertex energy, we find that configurations $\vec{\rho}$ that violate the linear constraints Supplementary Equation 4, i.e., $C_a^i \rho_i \neq 0$, are characterized by an energy $E \sim \rho^2$. On the other hand, configurations $\boldsymbol{\rho}$ in the $2d$ linearized null space satisfy $C_a^i \rho_i = 0$, thus their energy scales as $E \sim \rho^4$ [11] (The two special zero-energy motions of the vertex satisfy the constraints to all orders in ρ).

These two distinct scaling laws allow us to avoid the flat state bifurcation by lifting one of the zero-modes, in a similar manner to the way it was done for the 4-bar linkage. If the creases themselves have a heterogeneous profile of stiffness (or bending rigidity), some folding configurations can be made energetically favorable compared to others. This last notion is encoded in the energy model of the vertex

$$E = E_{\text{Vertex}} + E_{\text{Crease}} = \kappa_{\text{f}} T_a^2 + \frac{1}{2} \sum_{i \in \text{creases}} \kappa_i \rho_i^2, \tag{6}$$

with κ_i the stiffness of crease i. In practical applications, the folding stiffness moduli of the creases κ_i will depend on each crease's thickness, length and material properties. Generally the length and material are given by the type of application, so that the value of κ_i can be set by proper choice of the crease thickness t. It is known that for general elastic materials $\kappa \sim t^3$ [12].

Supplementary Figure 3 shows how changing the crease stiffness profile κ_i allows us to choose which of the two modes is lifted near the flat state, while the scale of the crease stiffness sets the folding value $\rho_{\text{c}} \sim \sqrt{\tilde{\kappa}_i / \kappa_{\text{f}}}$ in which the lifted mode returns via a saddle-node bifurcation. As we had for the 4-bar linkage, the linearized null space is spanned by two vectors $\boldsymbol{v}_1, \boldsymbol{v}_2$, such that configurations in the null space are given by

$$\boldsymbol{\rho} = \rho(\boldsymbol{v}_1 \sin\phi + \boldsymbol{v}_2 \cos\phi).$$

Large patterns with loops

When 4-vertices form a closed loop, an extra constraint is defined by the requirement that folding angles are consistent around each loop [13]. Just as for the independent vertex constraints, the extra constraint can be broken at the expense of bending the stiff face encompassed by the loop. We model this bending by adding a stiff 'face' crease inside each loop of vertices [11]. Since both vertex and loop constraints are broken by bending the same stiff faces, the bending modulus is the same κ_{f}. Together with the heterogeneous stiff creases defined in the main text, the origami energy model is given by [6–9]

$$\begin{aligned} E_{\rm sheet}(\boldsymbol{\rho}) &\equiv E_{\rm Vertex} + E_{\rm Loop} + E_{\rm Crease} \\ &= \kappa_{\rm f} \sum_a T_a^2 + \frac{1}{2}\kappa_{\rm f} \sum_{f \in \rm faces} \rho_f^2 + \frac{1}{2} \sum_{i \in \rm creases} \kappa_i \rho_i^2, \end{aligned} \tag{7}$$

We note that in the linearized null space about the flat state, both the vertex and loop terms scale as ρ^4 , while the crease bending term generically scales as ρ^2. Thus, at small folding magnitudes $\rho \ll \rho_{\rm c} \sim \sqrt{\bar{\kappa}_i/\kappa_{\rm f}}$ bending of creases is expensive and suppressed - minimizing the convex quadratic energy term. In contrast, at large folding magnitudes $\rho \gg \rho_{\rm c}$, face bending becomes expensive, such that all bending occurs at the creases. We conclude that by choosing a crease stiffness profile appropriately, the flat state branching can be avoided for arbitrarily large self-folding origami patterns.

Supplementary Note 3 - Folding methods

Patterns with stiff creases as defined above will settle to the flat state, the global minimal energy configuration of $E_{\rm sheet}$ (Supplementary Equation 7). When external folding torques $F_i^{\rm ext}(\rho, t)$ are applied to the creases i across the pattern, the sheet is folded away from the flat state by balancing the external forces and the internal forces due to face and crease bending (Supplementary Equation 7). We define folding through the equation,

$$\tau_{\rm relax} \frac{d\rho_i}{dt} = -\frac{\partial E_{\rm sheet}(\boldsymbol{\rho})}{\partial \rho_i} + F_i^{\rm ext}(\rho, t) \tag{8}$$

Here, $\tau_{\rm relax}$ is the folding relaxation timescale of the creases, determined by the elastic properties of the material [14]. This critical parameter sets the relevant timescale for all dynamics. For example, adiabatic folding implies the folding forces $F^{\rm ext}(t)$ change slowly relative to $\tau_{\rm relax}$ while fast folding implies $F^{\rm ext}(t)$ change fast relative to $\tau_{\rm relax}$.

In some cases, the folding forces can be captured by an energy function $E_{\rm folding}$, i.e., $F_i^{\rm ext}(\rho, t) = -\frac{\partial E_{\rm folding}(\boldsymbol{\rho})}{\partial \rho_i}$. In these select cases, we can write,

$$\tau_{\rm relax} \frac{d\rho_i}{dt} = -\frac{\partial (E_{\rm sheet}(\boldsymbol{\rho}) + E_{\rm folding}(\boldsymbol{\rho}))}{\partial \rho_i} \equiv -\frac{\partial E_{\rm tot}(\boldsymbol{\rho})}{\partial \rho_i}.$$

Torque-based folding

Several experiments [14] apply constant folding torques F_i to specific creases. One simple model of such folding experiments would be the following time-dependent energy,

$$E_{\text{tot}} = E_{\text{sheet}} - a(t) \sum_i F_i \rho_i \tag{9}$$

Here $a(t)$ describes a time-dependent protocol for ramping up the folding forces F_i. We take $a(t) = vt$, where v measures the speed of folding.

Simulations: We simulated folding using Supplementary Equation 9 but without stiff creases and find no success unless F_i has high dot product with the desired mode (Main text Figure 4(a)). It is hard to call high dot-product based folding 'self-folding' since such actuation requires a large number of actuator creases, and the torques F_i on each crease need to be tuned carefully.

However, with stiff creases, the 16 vertex pattern folds with large success rates for substantially smaller dot products. For example, a dot product of 0.2 suffices to fold the pattern correctly for $\sim 80\%$ of the patterns and forces sampled. The data shown is averaged over 5 patterns and 20 different random torque vectors F_i (for each sample and dot product), folded using a sufficiently slow protocol $a(t)$.

Topologically protected bifurcations:

Main text Figure 4(a) shows that sheets with stiff creases have dramatically higher success rate in folding using specific F_i, even though the crease stiffness profiles were chosen without any knowledge of these folding forces. We can understand the reason for success mathematically - our stiff crease prescription attempts to program the bifurcation diagram to eliminate misfolding pathways. Such topological properties of bifurcation diagrams are robust to the kinds of deformations induced by these folding forces.

The folding forces in Supplementary Equation 9 above modify the energy by a linear term $\sum_i F_i \rho_i$. Such modification cannot create new minima near the flat state since E_{sheet} has a saddle-node bifurcation only at a finite ρ_c.

To understand this first in a simple example, consider the function $f(x) + \lambda x$ where $f(x)$ has one unique minimum at some point x_0. In addition to the minimum at x_0, if $f(x)$

was posed at a saddle-node bifurcation at some point x_1 (e.g., $f(x) \sim (x - x_1)^3$ near x_1), then $f(x) + \lambda x$ will develop a new minimum at x_1 for infinitesimal λ. Instead, if $f(x)$ were some finite distance away from a saddle-node bifurcation in a neighborhood of x_1, we are guaranteed that no new minima are created by small enough λ.

In analogy, our design principles eliminated all undesired minima in E_{sheet} at saddle-node bifurcations at some finite distance ρ_{c} away from the flat state. Thus adding the linear term $\sum_i F_i \rho_i$ cannot create new minima in this landscape close enough to the flat state. Thus we can mathematically anticipate the significant success seen in main text Figure 4(a) with slow folding.

Target angle-based folding

A related method of folding involves target angles ρ_i^{target} for different creases. Such a target-based folding can be modeled by springs with rest angles at ρ_i^{target} that are 'turned on' by some actuation method (e.g., temperature, light changes [14, 15]),

$$E_{\text{tot}} = E_{\text{sheet}} + \frac{1}{2} \sum_i \eta_i (\rho_i - a(t)\rho_i^{\text{target}})^2 \tag{10}$$

where $a(t)$ describes the time-dependent protocol by which such creases are actuated; $a(t) = 0$ before actuation and the target angles are ramped up to final target ρ_i^{target}. We take $a(t) = vt$ and simulate slow folding. η_i is the strength of the springs with target angles; only a select set of creases are actuated in this manner ($\eta_i \neq 0$).

Simulations: we applied the folding method of Supplementary Equation 10 to 16 vertex patterns. This time, instead of varying the dot product between external forcing and the desired mode, we vary the number of creases that are actuated using target angle folding (the creases chosen are those who fold most in the desired mode $\boldsymbol{\rho}_D$). When the creases are soft, we again see that this model of folding can rarely find the desired mode (main text Figure 4(b)), even if a few creases are actuated simultaneously. On the other hand, applying the LP stiffness profile to the creases improves the success rate dramatically, even while actuating just 1 or 2 creases (data is averaged over 10 patterns).

Topologically protected bifurcations:

Sheets with designed stiff creases show high success rates with this mode of folding as well. Again, we could have anticipated this success mathematically. Note that Supplementary Equation 10 can be expanded and written as,

$$E_{\text{tot}} \sim E_{\text{sheet}} + \frac{1}{2}\sum_i \eta_i \rho_i^2 - a(t)\sum_i \eta_i \rho_i^{\text{target}} \rho_i$$

where we have dropped a term independent of ρ_i. The linear term $a(t)\sum \rho_i^{\text{target}}\rho_i$ is mathematically identical to the linear term in torque-based folding with $F_i = \eta_i \rho_i^{\text{target}}$. Hence folding can be expected to succeed for the same reasons.

However, the quadratic term $\eta_i \rho_i^2$ resembles an additional stiffness for the creases about the flat state, in addition to the designed stiffness κ_i present in E_{sheet}. Such extra stiffness could potentially be problematic. To see this, note that in the main paper, the designed stiffness κ_i were carefully chosen to satisfy a linear lifting condition (or saddle-node bifurcation condition) of the form,

$$\sum_{i \in \text{creases}} R_i \kappa_i \geq E_{\text{TS}}(\rho_{\text{c}}) \tag{11}$$

where $R_i \equiv \frac{1}{2}\rho_{\text{c}}^2\left[(\tilde{\rho}_{\text{U}})_i^2 - (\tilde{\rho}_{\text{TS}})_i^2\right]$ and $\tilde{\boldsymbol{\rho}}_{\text{U}}, \tilde{\boldsymbol{\rho}}_{\text{TS}}, \rho_{\text{c}}, E_{\text{TS}}$ were defined in the main text.

Shifting a solution κ_i of the above equation by $\kappa_i + \eta_i$ could, in principle, violate the above equation. The simulation results in main text Figure 4(b) show that such violations do not occur often for random patterns. However, when such violation do occur, they would result in undesired minima reappearing.

If η_i are known at the time of design, we can simply account for them in the above lifting constraint and use the following constraint instead,

$$\sum_{i \in \text{creases}} R_i \kappa_i \geq E_{\text{TS}}(\rho_{\text{c}}) - \sum_{i \in \text{creases}} R_i \eta_i \tag{12}$$

where $R_i \equiv \frac{1}{2}\rho_{\text{c}}^2\left[(\tilde{\rho}_{\text{U}})_i^2 - (\tilde{\rho}_{\text{TS}})_i^2\right]$.

To summarize, our crease stiffness prescription is designed to solve the misfolding problem for general. Thus we do not account for specific forces in the procedure to determine optimal crease stiffness profiles. In addition to the folding results shown in the main paper, the simulation results in main text Figure 4 show that the predicted stiffness profiles work for a range of folding forces and models, even if the forces were not known at design time. On the

other hand, Supplementary Equation 12 shows how specific folding forces and methods can be accounted for at design time to change failed cases into successfully folding protocols.

Strain-based folding

In some methods of folding [14], the sheet is generally compressed without specific forces F_i applied to specific creases. Without crease stiffness, such folding without specific forces is almost certain to misfold. We tested the folding success of such folding methods in patterns with stiff creases in simulation.

Patterns are initially set at a small folding magnitude $\rho \ll \rho_\mathrm{c}$, and allowed to relax (with fixed ρ) influenced by the sheet potential. As argued above, we find that when heterogeneous crease stiffness profiles are present, there exists a unique minimum at fixed small ρ (up to Z_2 symmetry). The pattern is folded with a strain based algorithm, such that the external torques applied to the pattern are proportional to the folding angles of the configuration. That is, we use an energy,

$$E = E_\mathrm{sheet} - a(t) \sum_i F_i \rho_i, \quad F_i = \frac{1}{2}\rho_i(t) \tag{13}$$

In practice, folding the pattern at a specified rate is achieved by an iterative two step algorithm: 1) Increase ρ by a specified amount $\Delta\rho$, keeping the direction $\vec{\rho}$ fixed, 2) Relax the configuration using the origami energy model (keeping ρ fixed).

While the first step in each iteration increases the magnitude of folding, the second step modifies the configuration in the angular directions by gradient descent, finding the nearest angular minimum. The slow folding limit is obtained by setting $\Delta\rho \ll \rho_\mathrm{c}$ in step 1, so that the one-dimensional tracks are followed adiabatically. The folding algorithm is implemented using MATLAB constrained optimization functionality. Results on the success of strain based folding methods with heterogeneous crease stiffness profiles are given in main text Figure 3.

Supplementary Note 4 - Crease stiffness selection

In the main text we present general arguments for the choice of crease stiffness profiles. Designed motions in large self-folding origami patterns can be approximately decomposed

to individual 4-vertices making a binary choice between their two zero-modes. This idea suggests that the designed motion can be targeted for adiabatic folding by lifting the undesired motion in each 4-vertex independently. The argument gives rise to a set of linear constraints lifting the unwanted minimum at a specific chosen ρ_c value. Together with a positivity constraint, these two inequality constraints limit the choice of stiffness profile, but maintain much of the stiffness design freedom. We note that the simple structure of the constraints guarantees the existence of a large feasible solution space, as the constraints ask for semi-positive $\boldsymbol{\kappa}$ vectors that exist above the hyper-plane defined by Equation 2 of the main text.

Although any random stiffness profile satisfying the inequality constraints will keep only the minimum related to the desired minimum, we further discuss how the design freedom can be utilized to minimize the energetic or geometric distortion of the resulting motion. These considerations give rise to the Linear and Quadratic Programming methods for selecting the stiffness profile.

Optimizing the energetic distortion of the desired mode ρ_{D} is performed by minimization of its energy due to the stiff creases:

$$E_{\mathrm{Crease}}(\boldsymbol{\rho}_{\mathrm{D}}) = \frac{1}{2} \sum_{i \in \mathrm{creases}} \kappa_i (\rho_{\mathrm{D}})_i^2 \tag{14}$$

This function is manifestly linear in κ_i, such that minimizing it (subject to the linear constraints) can be easily performed performed using Linear Programming, an efficient polynomial algorithm. As the linear optimization function generically looks for solution with small κ values, we find that this algorithm obtains solutions that saturate the lifting constraint of main text Equation 2.

Geometric distortion involves changing the minimal energy configurations due to the crease stiffness. We find this kind of distortion is present in every case that the creases of an origami pattern are made stiffer in a heterogeneous manner. If one wishes to design a pattern to have a specific precise folded state, it might be important to control geometric distortions due to stiff creases. To reduce the geometric distortion in a folded state we can optimize the energy due to stiff creases such that its angular minimum (energy minimum at given ρ) is close to the desired folded state. The gradient of crease energy at the desired mode configuration is:

$$\frac{\partial E}{\partial \rho_i}(\boldsymbol{\rho} = \boldsymbol{\rho}_{\rm D}) = (\boldsymbol{\kappa} \star \boldsymbol{\rho}_{\rm D})_i, \tag{15}$$

where $\star$ indicates element wise multiplication. If $\rho_{\rm D}$ is an exact angular minimum of the energy function, we know that the normalized dot product between $\frac{\partial E}{\partial \rho_i}$ and $(\rho_{\rm D})_i$ has to be unity, as the angular components of the gradient vanish and the force is entirely in the $\rho_{\rm D}$ direction:

$$\frac{\frac{\partial E}{\partial \rho_i}(\boldsymbol{\rho} = \boldsymbol{\rho}_{\rm D}) \cdot (\rho_{\rm D})_i}{||\frac{\partial E}{\partial \rho_i}(\boldsymbol{\rho} = \boldsymbol{\rho}_{\rm D})|| \cdot ||(\rho_{\rm D})_i||} = 1, \tag{16}$$

where the numerator multiplies the vectors $\rho_{\rm D}$ and the energy gradient at $\boldsymbol{\rho} = \boldsymbol{\rho}_{\rm D}$, while the denominator multiplies their norms. By writing these terms explicitly and rearranging them, we find that a configuration ρ_D that has no geometric distortion satisfies

$$\rho_{\rm D}^2 \sum_{i \in \text{creases}} \kappa_i^2 (\rho_{\rm D})_i^2 \; - \sum_{i,j \in \text{creases}} \kappa_i \kappa_j (\rho_{\rm D})_i^2 (\rho_{\rm D})_j^2 = 0 \tag{17}$$

Evidently, a solution for which this condition is satisfied is $\kappa_i = \text{const}$. Unfortunately, a homogeneous crease stiffness profile does not lift any of the modes near the flat state. We would like to find stiffness profiles that are heterogeneous to lift all but one minimum, but still optimize geometric distortion. This can be done if instead of demanding the condition of Supplementary Equation 17 be satisfied, we treat it as a function to be minimized subject to the linear constraints:

$$F_{\rm QP} \equiv \rho_{\rm D}^2 \sum_{i \in \text{creases}} \kappa_i^2 (\rho_{\rm D})_i^2 \; - \sum_{i,j \in \text{creases}} \kappa_i \kappa_j (\rho_{\rm D})_i^2 (\rho_{\rm D})_j^2 \tag{18}$$

Crucially, the function $F_{\rm QP}$ is manifestly quadratic in the stiffness profile κ_i, and positive semi-definite. These properties allow us to optimize $F_{\rm QP}$ with Quadratic Programming, another efficient polynomial algorithm. We use MATLAB Linear and Quadratic Programming routines to find these stiffness profile solutions. The main text shows how these schemes optimize energetic and geometric distortions of the chosen vertex motion.

For single 4-vertices, one could sample random stiffness profiles that satisfy the linear constraints and come close to optimizing mode distortions. The major gains of using our stiffness optimization protocols manifest when considering larger patterns. As shown in Supplementary Figure 4(a), even for simple looped patterns (quads), LP and QP stiffness

protocols achieve optimized distortion values better by orders of magnitude compared to other schemes, including the $\boldsymbol{\rho}_{\mathrm{D}}^{-n}$ prescription that worked well for single vertices. The superior performance of the optimized protocols was established over a sample of 100 patterns.

For larger patterns still, random stiffness profiles that satisfy the linear constraints are usually accompanied by very high energetic and geometric distortions. Our optimized stiffness selection protocols are superior to any other choice, yet are themselves subject to degradation for large patterns. Supplementary Figure 4(b-c), shows how the optimum energetic and geometric distortions (achieved by LP and QP, respectively) grow with pattern size.

Finally, we tested the robustness of our method to manufacturing errors in the stiffness profile. We simulated such errors in small and large patterns by perturbing the optimal solutions (obtained by either LP or QP) with relative values of up to 5%. We find that the resulting unique low-ρ mode is essentially a perturbed version of the one obtained for the optimal solution. The fact that small perturbations in κ lead to small distortion of the unique low-ρ mode means that our method is expected to be robust to manufacturing errors in the stiffness profile κ.

Supplementary Note 5 - Adiabatic failures and bifurcations along the desired pathway

The main text discusses how selecting an appropriate crease stiffness profile establishes a smooth folding motion connecting a unique minimum close to the flat state and a desired nonlinear folded configuration. Indeed, we find that the designed folded configuration frequently connects continuously to the unique small ρ minimum established by stiffness profiles due to LP or QP springs. This gives rise to the heuristic approach of folding self-folding origami: Design the pattern with a heterogeneous crease stiffness profile (solving LP or QP), and fold the pattern slowly.

Although this idea appears to work for many crease patterns ($> 90\%$ of the 3×3 patterns in our sample), it is not universally successful. In some patterns (Supplementary Figure 5) we observe that the desired folded configuration does not connect continuously to the unique low-ρ minimum, but instead terminates at a saddle-node bifurcation. In turn, the unique

low-ρ minimum itself is also terminated with a saddle node-bifurcation. Thus, failure to fold adiabatically is associated with a saddle-node bifurcation along the desired pathway.

Such failures can be mathematically ruled out if the states along adiabatic pathway are always the lowest energy state for each ρ since ground states cannot undergo saddle-node bifurcations. In fact, we know that the pathway has lowest energy at low and high ρ — there is only one state at low ρ and at high, the pathway connects with the desired mode which is designed to be the lowest energy state by solving loop equations [13]. However, such protection does not extend to intermediate ρ. If a lower energy state exists at finite ρ, the desired pathway could undergo a saddle-node bifurcation like that shown in Supplementary Figure 5, preventing adiabatic folding.

In the particular case presented here, the likely candidate is a distractor configuration shown in blue. When this distractor folded state is unfolded slowly, it morphs into a state that has smaller energy than the unique low-ρ minimum. This allows the unique low-ρ minimum to terminate in a saddle-node bifurcation. When folded slowly from the flat state, the adiabatic pathway described by the unique low-ρ minimum abruptly terminates, and the folded state snaps into another configuration, which itself adiabatically morphs into a distractor mode.

Supplementary References

[1] Richard Scheunemann Hartenberg and Jacques Denavit. *Kinematic synthesis of linkages*. McGraw-Hill, 1964.

[2] J Michael McCarthy. *Geometric design of linkages*, volume 11. Springer Science & Business Media, 2006.

[3] D Rocklin, Vincenzo Vitelli, and Xiaoming Mao. Folding mechanisms at finite temperature. *Preprint at http://arXiv.org/abs/1802.02704*, 2018.

[4] Tomohiro Tachi. Geometric considerations for the design of rigid origami structures. In *Proc. Int. Assoc. Shell Spatial Struct. Symp.*, volume 12, pages 458–460, 2010.

[5] Sarah-Marie Belcastro and Thomas C Hull. Modelling the folding of paper into three dimen-

sions using affine transformations. *Linear Algebra Appl.*, 348(1):273–282, 2002.

[6] Tomohiro Tachi. Design of infinitesimally and finitely flexible origami based on reciprocal figures. *J. Geom. Graph.*, 16(2):223–234, 2012.

[7] Arthur A Evans, Jesse L Silverberg, and Christian D Santangelo. Lattice mechanics of origami tessellations. *Phys. Rev. E*, 92(1):013205, 2015.

[8] Levi H Dudte, Etienne Vouga, Tomohiro Tachi, and L Mahadevan. Programming curvature using origami tessellations. *Nat. Mater.*, 15:583–588, 2016.

[9] Bryan Gin-ge Chen and Christian D Santangelo. Branches of triangulated origami near the unfolded state. *Phys. Rev. X*, 8(1):011034, 2018.

[10] Alex Lobkovsky, Sharon Gentges, Hao Li, David Morse, and Thomas A Witten. Scaling properties of stretching ridges in a crumpled elastic sheet. *Science*, 270(5241):1482–1485, 1995.

[11] Menachem Stern, Matthew B Pinson, and Arvind Murugan. The complexity of folding self-folding origami. *Phys. Rev. X*, 7(4):041070, 2017.

[12] Thomas A Witten. Stress focusing in elastic sheets. *Rev. Mod. Phys.*, 79(2):643, 2007.

[13] Matthew B Pinson, Menachem Stern, Alexandra Carruthers Ferrero, Thomas A Witten, Elizabeth Chen, and Arvind Murugan. Self-folding origami at any energy scale. *Nat. Commun.*, 8:15477, 2017.

[14] Edwin A Peraza-Hernandez, Darren J Hartl, Richard J Malak, Jr, and Dimitris C Lagoudas. Origami-inspired active structures: a synthesis and review. *Smart Mater. Struct.*, 23(9):094001, 2014.

[15] Jesse L Silverberg, Jun-Hee Na, Arthur A Evans, Bin Liu, Thomas C Hull, Christian D Santangelo, Robert J Lang, Ryan C Hayward, and Itai Cohen. Origami structures with a critical transition to bistability arising from hidden degrees of freedom. *Nat. Mater.*, 14(4):389–393, 2015.
