# Supplementary material for: Shaping the topology of folding pathways in mechanical systems
Source: Nat Commun. 2018 Oct 16;9:4303. doi: 10.1038/s41467-018-06720-1 (PMC6191449; doi:10.1038/s41467-018-06720-1)
Supplement: Supplementary file 3 — Description of Additional Supplementary Files [file 41467_2018_6720_MOESM3_ESM.pdf]

Description of Additional Supplementary Files

File Name: Supplementary Software 1
Description: CreasePattern.m - class, definitions, and folding functions for origami patterns.

File Name: Supplementary Software 2
Description: fnm compute CF.m - computes the vertex energy at a given folded configuration.

File Name: Supplementary Software 3
Description: PartialPattern.m - divides large crease patterns to individual vertices.

File Name: Supplementary Software 4
Description: StiffnessLP.m - finds the optimal stiffness profile using the Linear Programming (LP) method.

File Name: Supplementary Software 5
Description: StiffnessQP.m - finds the optimal stiffness profile using the Quadratic Programming (QP) method
